# Forgotten and ignored: making digital health work for migrant population in Africa

**DOI:** 10.1093/oodh/oqae023

**Published:** 2024-07-23

**Authors:** Taofeekat Adigun, Esther Opone, Bettina Baidoo, Moses Mathenge, Cephas Avoka, Olutola Awosiku

**Affiliations:** Sheffield Centre for Health and Related Research, University of Sheffield, 30 Regent St, Sheffield City Centre, Sheffield S1 4DA, UK; Department of Biomedical Laboratory Science, University of Ibadan, Nigeria, Box 4078, University of Ibadan Post, Ibadan 200001, Oyo State, Nigeria; Department of Medicine, College of Medicine, University of Cape Town, School of Public Health Barnard Fuller Building, Anzio Rd, Observatory, Cape Town 7935, South Africa; Department of Health Science, Kenyatta University, School of Health Sciences Off Thika Rd, 43844-00100 GPO, Nairobi, Kenya; Ghana College of Physicians and Surgeons1 Jomo Kenyatta Roade, Ridge, Accra, P. O. Box MB 429, Accra, Ghana; Department of Biomedical Laboratory Science, University of Ibadan, Nigeria, Box 4078, University of Ibadan Post, Ibadan 200001, Oyo State, Nigeria

**Keywords:** digital health, migrant population, Africa, universal health coverage, healthcare disparities, healthcare access

## Abstract

The African continent has experienced an alarming increase in forcibly displaced individuals, driven by socio-political conflict, economic instability and climate-induced calamities. The urgent need for contextualized and adaptable health solutions in the face of ongoing conflicts and crises underscores the importance of harnessing digital health innovations while ensuring inclusivity and equity for all. This commentary explores the potential of digital health to address healthcare disparities among migrant populations, examining the barriers to adoption and providing recommendations for policymakers and stakeholders to promote inclusivity and improve healthcare access. While digital health emerged as a promising avenue for improving healthcare access, there is also a greater necessity for tailoring these innovations to the specific needs and vulnerabilities of the target populations.

## INTRODUCTION

The 21st century witnessed an unprecedented surge in forced displacement, with the African continent being the focal epicentre of this global crisis. Data from the United Nations High Commissioner for Refugees (UNHCR) indicates a rise of 2.3 million forcibly displaced individuals in 2018, culminating in an alarming aggregate exceeding 70 million by the close of the year [1]^.^ In Africa, this mass displacement is triggered by a confluence of factors, including socio-political conflict, economic instability and the pernicious effects of climate change [1, 2]. Notably, climate-induced calamities such as severe drought and famine have become potent drivers of migration, disproportionately affecting the continent’s most vulnerable populace [3]. This trajectory has consequently propelled the total population of concern (PoC) to the UNHCR in Africa to a staggering 26.4 million in 2018, encompassing various categories including internally displaced persons (IDPs), asylum seekers, stateless individuals, returnees and others [1].

The collective of migrant communities constitutes a diverse spectrum, spanning from refugees crossing international borders and both documented and undocumented migrants to internally displaced people (IDPs), each grappling with distinct and pressing challenges. Unlike refugees, IDPs do not cross international borders; rather, they are compelled to vacate their habitual residences because of conflicts, environmental upheavals or climate disasters [4]. This predicament exposes them to a plethora of human rights violations and health disparities, often surpassing those of other migrant subgroups. While humanitarian aid ostensibly extends respite and assistance to IDPs, it inadvertently engenders an environment of dependency, impeding their autonomy and circumscribing their agency in determining the nature of the care they receive [5]. The magnitude of migration across the African terrain exerts substantial pressure on healthcare infrastructure, exacerbating preexisting deficiencies in healthcare access and delivery. Notably, structural challenges, such as a dearth of trained medical personnel, further reduce access to healthcare services [3]. Furthermore, the healthcare infrastructure is woefully inadequate for host communities, and this inadequacy is worse for displaced persons with longer wait times for public health services, delayed treatment and a shortage or unavailability of medication. Adding to the complexity of the situation is an often neglected issue of mental health, which presents a vital concern for populations traumatized by the aftermath of war, violence or forced displacement [2, 6]. Amidst this bleak landscape, the transformative potential of digital health presents an exciting opportunity for innovation and improvement in healthcare delivery. However, ensuring digital health equity is a foundational prerequisite for harnessing this potential. Digital health equity is an equitable opportunity for individuals to benefit from knowledge and practices intrinsic to the development and utilization of digital technologies to improve health [7]. It is imperative to note that inequities in society often reflect in digital health equity; hence, those at risk of social exclusion due to low education or precarious economic circumstances face heightened susceptibility to exclusion from digital health services [8]. Digital health has the potential to provide low-cost solutions for expanding information and access to health services for migrant populations. In this commentary, we examine the critical role that digital health innovations can play in enabling access to healthcare services for migrant/displaced populations and highlight contextual barriers, policy ramifications and ethical considerations influencing the accessibility and utilization of these digital health innovations. Finally, we propose recommendations to address the healthcare needs of migrant and displaced populations.

### The role of digital health in advancing healthcare access for migrant populations

The African Union’s bold vision in its 2018–2027 migration policy framework underscores the importance of healthcare access for migrants [9]. Yet, the multifaceted challenges posed by pervasive resource constraints across many African nations continue to hinder the integration of migrants and refugees into healthcare systems. This predicament is further compounded by the social exclusion and uphill battle faced by a significant proportion of the migrant population. Against this backdrop, the search for improved migrant health has prompted the consideration of digital tools as a transformative solution within the humanitarian health domain [10]. By encompassing a broad range of information and communication technologies, digital health seeks to prevent and manage diseases and enhance well-being using e-health, mobile health, telehealth and health information technology [11]. Due to their cost-effectiveness and scalability, these tools present an array of solutions to overcome the exigent health challenges precipitated by humanitarian crises. From telemedicine to mental and psychosocial support, these tools not only bridge the healthcare gap but also empower migrants to actively seek customized healthcare solutions aligned with their distinct needs rather than relying solely on externally provided aid. Embedded within digital health and humanitarian efforts is an avenue for addressing the pronounced vulnerabilities and social exclusion endemic to healthcare access within migrant communities [12]. However, this convergence is not without its pitfalls. Digital health tools that are ill-suited or contextually misaligned may inadvertently exacerbate existing vulnerabilities and inequalities, particularly when they fail to harmonize with migrants’ unique needs. The equity and accessibility of digital health technologies, including devices and connectivity, are unequal and further compounded by the poor digital maturity in Africa. This further highlights the marginalization that these technologies may pose to vulnerable populations in the region [13]. The crucial undertaking lies in meticulously tailoring these tools to provide culturally competent healthcare services and timely interventions that are sensitive to migrants’ unique challenges [14].

The urgency to integrate and amplify digital health tools has become even more palpable, given the rising conflicts and political upheavals across the continent. Recent events, such as the devastating conflict in Sudan and multiple military coups, have intensified the precarious circumstances of countless migrants [15]. These ongoing battles and shifts in power have led to massive population displacement, with many finding themselves in perilous situations with limited access to essential health services. In such contexts of heightened instability and unpredictability, it becomes imperative to find agile and adaptable health solutions that cater to these vulnerable populations. However, digital health technologies and other applications of ICTs should be guided by a systemic, social and collective approach that creates the best practices and, on the other hand, is efficient for use by vulnerable groups.

### Barriers to digital health adoption among migrants and refugees

Research has underscored that migrants and ethnic minorities are less inclined to search for health-related information online than the general population, with a migrant background being associated with a lower understanding of web-based health information [16, 17]. Key barriers to digital health service access among migrants include insufficient digital and language skills [18, 19]. The use of these services requires not only proficiency in the local language but also a command of specific administrative and medical vocabulary. Further barriers to adoption persist significantly, including factors, such as computer literacy, financial constraints, data security and inadequate technological infrastructure [17, 18]. Lawal et al. [20] found that language barriers were among the limitations faced by refugees when accessing digital health services in an IDP camp in Nigeria. Inadequate digital literacy has emerged as a pronounced hindrance to adopting digital health solutions [16, 20]. This unfamiliarity with digital devices, software and applications impedes the adept use of digital health solutions. While younger generations might be more tech-savvy, older individuals and those with limited education may struggle to navigate these technologies, ranging from health applications, telemedicine platforms, online medical resources or other digital health tools, resulting in the underutilization or mismanagement of these resources [21].

Many studies on the design and implementation of digital technologies indicate a lack of cultural sensitivity, specifically in the African context. These biases arise from the data being trained and not tested with vulnerable individuals, such as refugees and migrants, and may perpetuate some existing inequalities [22]. These biases also raise concerns about the nature of some of these technologies in providing inclusive healthcare for all Africans. Concurrently, constrained access to technology and Internet infrastructure impedes the adoption of digital health among migrants and refugees across Africa. Settlement patterns often lead migrants and refugees to distant, resource-constrained areas, where access to digital infrastructure, such as reliable Internet connectivity, electricity or even basic mobile phones, is limited [23]. Remarkably, Africa’s Internet penetration rate, according to Internet World Stats (2022), is the lowest at 43.2%, which is significantly lower than the global average of 73.55%. This digital divide disproportionately affects marginalized communities, including migrants and displaced individuals, limiting their ability to access and utilize digital health services effectively and benefit from them. Apprehensions concerning the privacy and security of personal health information increase hesitancy among migrants and refugees to adopt digital health solutions, particularly given their vulnerability and apprehensions of discrimination or persecution [24]. The latent apprehension that their data might be misappropriated or accessed without authorization could lead to unease and erode trust in digital health platforms [16]. In some regions, the legal and regulatory landscape may be unconducive to the integration of digital health technologies for migrants and refugees. Ambiguous and stringent policies in some countries regarding the use of digital health technology by migrants and refugees render access to adequate healthcare support onerous. For example, digital health platforms may require formal identification or residency documentation, which many migrants and refugees lack and have lost in the course of displacement.

A byproduct of recurrent displacement and migration surfaces in the loss of tangible health records, crucial for ensuring continuous and appropriate care. As people move between countries or regions, their health information may not be easily accessible or transferable, culminating in suboptimal healthcare outcomes and a lack of appropriate follow-up care. Refugees often experience difficulties in accessing their medical records and continuity of care owing to mobility-related challenges [25]. Digital health systems can mitigate this issue by providing accessible electronic health records regardless of geographical location. However, issues, such as interoperability and connectivity warrant attention to ensure the efficacy of these solutions. The absence of standardized procedures and compatibility between different health systems and platforms can result in fragmented health records, impeding stakeholders’ access to health information [26]. This leads to an incomplete medical history, potential medical errors and gaps in treatment and healthcare services, hampering the continuity of care. Interoperability challenges have been acknowledged as a significant concern in the global digital health landscape [27].

Understanding the barriers to digital health adoption among migrants and refugees across Africa is an indispensable step towards devising targeted strategies that cater to their distinct needs, thereby enriching healthcare accessibility and outcomes, and ultimately bolstering the realization of Universal Health Coverage for these populations.

### Recommendations to promote digital health inclusivity for migrants and refugees in Africa

While many obstacles to digital health adoption affect both migrants and host populations, the migrant population faces specific health needs and vulnerabilities that require culturally sensitive and effective digital health care that recognizes and responds to those needs. To ensure inclusive digital health, we recommend a two-pronged approach. First, addressing the particular challenges faced by migrant and refugee populations necessitates a comprehensive needs assessment. This assessment should include the distinct challenges faced by migrants, refugees and host populations in their respective contexts. Identifying areas where interventions can benefit both groups and where targeted solutions are necessary is essential. Developing low-tech digital health solutions that are specifically designed for use in resource settings common in humanitarian contexts. This may include short messaging services, offline-capable apps, low-bandwidth telemedicine platforms and energy-efficient devices that can operate in areas with limited electricity access, like refugee camps. We should also aim to integrate digital health initiatives into existing social welfare programmes and humanitarian services, including healthcare delivery, sexual health services, nutrition programmes and psychosocial support in refugee camps and migrant communities.

Second, it’s highly important to match digital health solutions to the maturity level of the ecosystem in which they are implemented. Some interventions may not be suitable for certain contexts until the digital health infrastructure and ecosystem have sufficiently advanced. Enhancing the digital maturity of African nations as a whole is crucial. This involves improving infrastructure, accessibility and digital literacy across the continent to support and sustain the implementation of digital health technologies.

Public-private partnerships can be leveraged to extend Internet coverage and provide affordable devices, making digital health services more accessible to populations as a whole. Examples include collaborating with telecommunication companies and NGOs to set up community-based Internet access points and provide affordable smartphones, devices and data plans for healthcare purposes. Enhancing digital resilience and maturity at the country and regional levels will ensure sustained access to digital health services for all populations, regardless of their status or location.

While short-term solutions are crucial for immediate response, considering longer-term strategies is equally vital. Exploring options for relaxing identification or residency requirements to access essential healthcare services can further promote digital health inclusion. Regular monitoring and evaluation of digital health initiatives must be conducted to assess their effectiveness and to identify areas for improvement. Data-driven insights will help policymakers and stakeholders refine their strategies and ensure continuous progress towards digital health inclusivity and long-term sustainability. Overcoming the barriers to digital health adoption will not only contribute to improved access and healthcare outcomes for vulnerable populations but also align with the broader goal of achieving Universal Health Coverage for all.

## Data Availability

No new data were generated or analyzed in support of this article.
